# A randomized controlled clinical trial on multimodal prehabilitation in colorectal cancer patients to improve functional capacity: preliminary results

**DOI:** 10.1007/s00464-024-11198-8

**Published:** 2024-08-29

**Authors:** Antonio Pesce, Nicolò Fabbri, Simona Colombari, Lisa Uccellatori, Giovanni Grazzi, Rosario Lordi, Gabriele Anania, Carlo Vittorio Feo

**Affiliations:** 1 Department of Surgery, Azienda USL of Ferrara and Azienda Ospedaliero Universitaria di Ferrara, University of Ferrara, Via Valle Oppio, 2, 44023 Lagosanto, Ferrara, Italy; 2 Clinical Nutrition, Azienda Ospedaliero Universitaria di Ferrara, Ferrara, Italy; 3 Division of Exercise and Sports Medicine, Department of Sports Medicine, Azienda USL di Ferrara, University of Ferrara, Ferrara, Italy

**Keywords:** Prehabilitation, Colorectal surgery, Functional capacity, 6-minute walking test, Post-operative outcomes

## Abstract

**Introduction:**

Major colorectal surgery is associated with 20 to 40% reduction in physiological and functional capacity and higher level of fatigue 6 to 8 weeks after surgery. The primary aim of this study was to analyse the effects of a multimodal prehabilitation program in colorectal cancer patients to improve functional capacity. The secondary outcome was to evaluate postoperative complications and length of postoperative hospital stay as well as to determine the costs of implementation and indirect costs.

**Methods:**

A single centre, single-blind, randomized controlled trial was conducted. Patients of age > 18 years undergoing elective colorectal resection for colonic cancer were eligible. Exclusion criteria were metastatic disease, severe walking impairments, renal failure stage > 2, ASA score > 3, preoperative chemo-radiation therapy. Patients have been randomized either to prehabilitation intervention groups, receiving 4-week trimodal prehabilitation (physical exercise and nutritional and psychological support) or to control receiving no prehabilitation. Both groups followed enhanced recovery programs and received rehabilitation accordingly. The primary outcome for functional capacity was measured by the 6-Minute Walking Test (6MWT) 4 and 8 weeks after surgery; to evaluate post-operative complications the Clavien-Dindo classification was used.

**Results:**

An interim analysis of 71 patients undergoing colorectal surgery was performed, with 35 assigned to interventional arm and 36 to control arm. Baseline characteristics were comparable in both groups. The prehabilitation group showed a significant increase in mean 6MWT distance pre-operatively compared to the control group, with an increase of 96 m (523 ± 24.6 vs. 427 ± 25.3, *p* = *0.01*). At 4 and 8 weeks, the prehabilitation group maintained significant improvements, with an increase of 103 m (514 ± 89 vs. 411 ± 115, *p* = *0.003*) and 90 m (531 ± 82 vs. 441 ± 107, *p* = *0.008*), respectively. There were no statistical significant differences in post-operative complications and hospital length of stay between the two groups.

**Conclusions:**

The preliminary results of this study indicate that it is feasible to implement a prehabilitation protocol lasting approximately 4 weeks. This protocol appears to yield a significant improvement in the physical performance of patients with colon cancer undergoing elective colorectal resection at 4 and 8 weeks after surgery.

**Graphical abstract:**

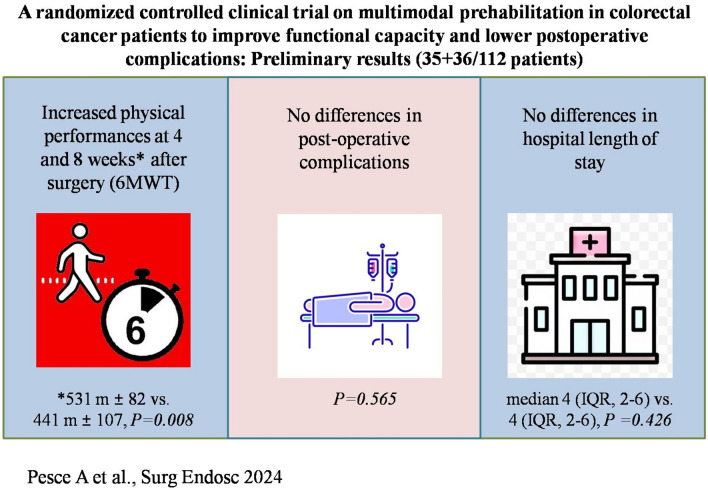

Postoperative complications occur in up to 50% of patients undergoing cancer colorectal resection and are associated with poor prognosis, increased costs, and lower health-related quality of life (HRQoL) [1–3]. Even with no complications, major surgery is associated with 20 to 40% reduction in physiological and functional capacity and higher levels of fatigue 6 to 8 weeks after surgery [4]. Many negative effects of major surgery can be reduced attenuating the surgical stress response by Enhanced Recovery After Surgery (ERAS) programs, to facilitate return of functional activities and accelerate convalescence [5].

In this study, we hypothesize that the implementation of a trimodal prehabilitation program (physical exercise, nutritional optimization, and psychological support) in patients undergoing ERAS colorectal resection for cancer with standard rehabilitation may determine: (1) better physical performance at 4 and 8 weeks after surgery, (2) decrease in postoperative complications and length of stay, and (3) reduced direct and indirect costs.

The primary aim was to compare the postoperative functional capacity in two groups of patients undergoing colorectal resection for cancer, one treated with 4-week trimodal prehabilitation before surgery followed by standard rehabilitation (prehabilitation group) and the other undergoing standard rehabilitation alone after surgery (control group). The secondary outcome was to evaluate postoperative complications and length of postoperative hospital stay as well as to determine the costs of implementation and indirect costs.

## Materials and methods

A single centre, single-blind, randomized controlled trial was conducted. Patients of age > 18 years undergoing elective colorectal resection for colonic cancer were eligible. Exclusion criteria were metastatic disease, severe walking impairments, renal failure stage > 2, ASA score > 3, preoperative chemo-radiation therapy. Patients have been randomized either to prehabilitation intervention group, receiving 4-week trimodal prehabilitation, or to control receiving no prehabilitation. Both groups received perioperative care and rehabilitation following current international ERAS guidelines [6]. The primary outcome for functional capacity was measured by the 6-Minute Walking Test (6MWT), chosen as a validated, objective measure of colorectal surgery recovery integrating all components of physical activity [7, 8]. Secondary criteria for primary outcome were: (a) cardiopulmonary exercise testing (CPET), (b) handgrip strength, (c) sit-to-stand.

All measures have been recorded in both study groups at baseline (beginning of prehabilitation period, 4 weeks before surgery; T -4) and prior to surgery (end of prehabilitation period; T 0) to measure the effects of intervention, and at 4 (T + 4) and 8 (T + 8) weeks after surgery to assess the impact of the intervention throughout the perioperative period. The investigator submitting the questionnaires and forms to the participants was not aware of the study hypothesis and she had no access to data.

Written informed consent was obtained from all eligible patients before inclusion in the study. The study followed the consolidated standards of reporting trials (CONSORT) guidelines [9]. The study protocol (ID: 849/2019/Sper/AUSLFe) was approved by the local Ethical Committee (*Comitato Etico Area Vasta Emilia Centro*– CE-AVEC) and was performed in accordance with the Declaration of Helsinki guidelines. The protocol of this randomized controlled trial is registered on ClinicalTrials.gov (Unique identifying number or registration ID: NCT06443203).

## Elements of trimodal prehabilitation

### Experimental group

#### Exercise program

A sports medicine physician assessed the mobility and exercise capacity of the patients. All participants underwent CPET, and the exercise intensity has been defined and personalized. Based on the assessment, the sports medicine physician prepared, explained, and coordinated a personalized program. The patients had three supervised exercise sessions per week that included interval and resistance training. They have been instructed on how to perform aerobic exercise at home (walking or cycling), initially at 50% of their calculated heart rate reserve with the goal of 60 min 4 times per week as tolerated by the patient. Accurate measurement of compliance is critical to determine the real effect of prehabilitation; therefore, the patients wore an accelerometer during the 4 weeks of intervention.

CPET is considered the gold standard, non-invasive measure to assess cardiorespiratory fitness and exercise capacity of an individual [10]. Patients underwent CPET using a maximal protocol based on incremental walking on a treadmill with increasing speed and incline. Patients were instructed not to consume any food or beverages (except water) at least 2 h before the test and to refrain from engaging in any form of physical exercise in the 2 days prior to the examination. Additionally, all subjects evaluated continued to take their necessary pharmacological therapy for the treatment of their conditions. The calculation is based on the determination of Oxygen-volume (VO2max) using the American Association of Cardiovascular and Pulmonary Rehabilitation protocol (AACVPR 2004) on a treadmill, which started at a speed of 2.4 km/h and 1.5% incline, with subsequent increments of 0.16 km/h and 0.5% incline every thirty seconds [11]. VO2max is a biomarker of health that measures the system’s ability to supply oxygen to the muscles. Many of the physiological markers that might lead to an increase in VO2max are improved by regular exercise.

The 6MWT is a commonly used test in clinical settings for the objective assessment of functional exercise capacity. This low-complexity and safe test typically involves asking the patient to walk along a 30-m corridor for a total of 6 min, aiming to cover the maximum distance possible during the test. The main parameter considered is the distance covered in the 6 min of walking, the so-called “6-Minute Walking Distance” (6MWD).

#### Nutritional supplements

The goal is to achieve an anabolic state in all patients, improving the amount of lean body mass in cachectic patients. At time zero, the dietitian assessed the patient’s nutritional status (body composition, caloric balance, and diet), according to the ESPEN Clinical Guidelines on Clinical Nutrition and Surgery [12]. Nutritional supplements have been adapted to the patient’s food intake. If there is weight loss or cachexia and an indication for extra nutrition without any medical limitations, the goal is to increase protein intake to 1.5 g/kg/day. In addition, 30 mg of whey protein 1 h after physical exercise and 1 h before bed time were also provided. Extra supplements such as multivitamins, one effervescent tablet a day for 2 weeks and omega-3 fatty acid from fish oil, two effervescent tablets a day for 2 weeks have been added.

#### Psychological coping

Patients awaiting cancer surgery may experience anxiety with symptoms of depression that could affect social and functional activities. Therefore, patients have been evaluated by a trained psycho-oncologist using validated questionnaires (Generalized Anxiety Disorder-7, GAD-7 and Patient Health Questionnaire-9, PHQ-9) [13]. The goal is to reduce anxiety and prevent depression by placing patients in a well-informed and active coping role to deal with the disease. If indicated (i.e., GAD-7 score ≥ 10 or PHQ-9 score ≥ 15), patients received a 90-min psychological intervention in the first session and additional sessions during the 4-week prehabilitation period. In the first preoperative session, for 60 min, the patient’s anxiety, coping strategies, and postoperative expectations have been addressed. The last 30 min were dedicated to teaching relaxation techniques, breathing exercises, and providing written instructions for practice at home.

A summary of the key elements of trimodal prehabilitation is shown in Table 1**.**

**Table 1:** Summary of the prehabilitation program

Component	Details
*Exercise program*	
Assessment	A sports medicine physician assesses mobility and exercise capacity and perform CPET to personalize exercise intensity
Supervised exercise	Patients have three supervised exercise sessions per week that include interval and resistance training
Home exercise	Patients has been instructed on how to perform aerobic exercise at home, starting at 50% of their calculated heart rate reserve, with a goal of 60 min 4 times per week
Compliance measurement	Patients wore an accelerometer during the 4-week intervention period
*Nutritional supplements*	
Assessment	A dietitian assessed the patient’s nutritional status, including body composition, caloric balance, and diet
Supplement adaptation	Nutritional supplements have been adapted to the patient’s food intake
Protein intake	If there is weight loss or cachexia, protein intake has been increased to 1.5 g/kg/day
Additional supplements	Multivitamins and omega-3 fatty acids from fish oil have been added for 2 weeks
*Psychological coping*	
Evaluation	Patients has been evaluated by a trained psycho-oncologist using validated questionnaires (GAD-7, PHQ-9)
Goal	To reduce anxiety and prevent depression by placing patients in a well-informed and active coping role to deal with the disease
Psychological intervention	If indicated, patients received a 90-min psychological intervention in the first session and additional sessions during the 4-week prehabilitation period
Preoperative session	In the first preoperative session, for 60 min, the patient’s anxiety, coping strategies, and postoperative expectations have been addressed
Relaxation techniques	The last 30 min have been dedicated to teaching relaxation techniques, breathing exercises, and providing written instructions for practice at home

### Control group

Patients have been encouraged to walk daily for at least 30 min, to perform breathing exercises for 5 min per day and 5–10 min of aerobic exercises. Psychometric analysis and nutritional assessment have been also performed and measured in the control group.

#### Secondary outcomes

To evaluate post-operative complications, the Clavien-Dindo classification has been used as a combined measure of morbidity and mortality [14].

Indicators of socio-economic characteristics, the perceived quality of life (Short Form-36, EORTC 29, 30), and patients' use of social and health care resources have been collected.

In order to determine indirect costs, a specific questionnaire was administered to all patients to evaluate: (1) the impact of surgery on productivity loss while carrying out daily activities; (2) the burden on caregivers; and (3) the working condition and the impact of surgery on working activity.

#### Statistical analysis

Patients have been randomized with a 1:1 allocation by means of randomization software. The sample size calculation was based on the primary aim, the 6MWT. We used preliminary data from a sample (*N* = 10) who underwent ERAS colonic resection for cancer, evaluated at baseline and after a 4-week prehabilitation program with 6MWT. Mean baseline walking capacity was 571.5 ± 64.1 m (m), while following the 4-week prehabilitation program increased to 618.8 ± 57.5 m with a mean change of + 47.2 ± 57.5 m. We hypothesized that 8 weeks after surgery the difference in the 6MWT between the prehabilitation and the control group would be about 45 m. Assuming a similar baseline walking capacity between the two study groups and an 8 weeks difference of about 45 ± 57 m, with an alpha level of 0.05 a power of 80%, the required sample size was 51 patients per group. Assuming a 10% attrition we planned to enroll 112 patients. Univariate analyses hasvebeen conducted to determine differences between groups on one time point with independent t-tests for continuous variables and chi-square analyses for categorical variables. Generalized mixed models for repeated measures have been used to analyze the effects of the prehabilitation program on several outcomes. Linear models were used for continuous outcomes (primary research question) and logistic models for dichotomous outcomes. Socio-demographic and clinical characteristics were also analyzed as time-invariant predictors. All data were analyzed using *STATA statistics* data analysis version 11.0.

## Results

**Fig. 1:**
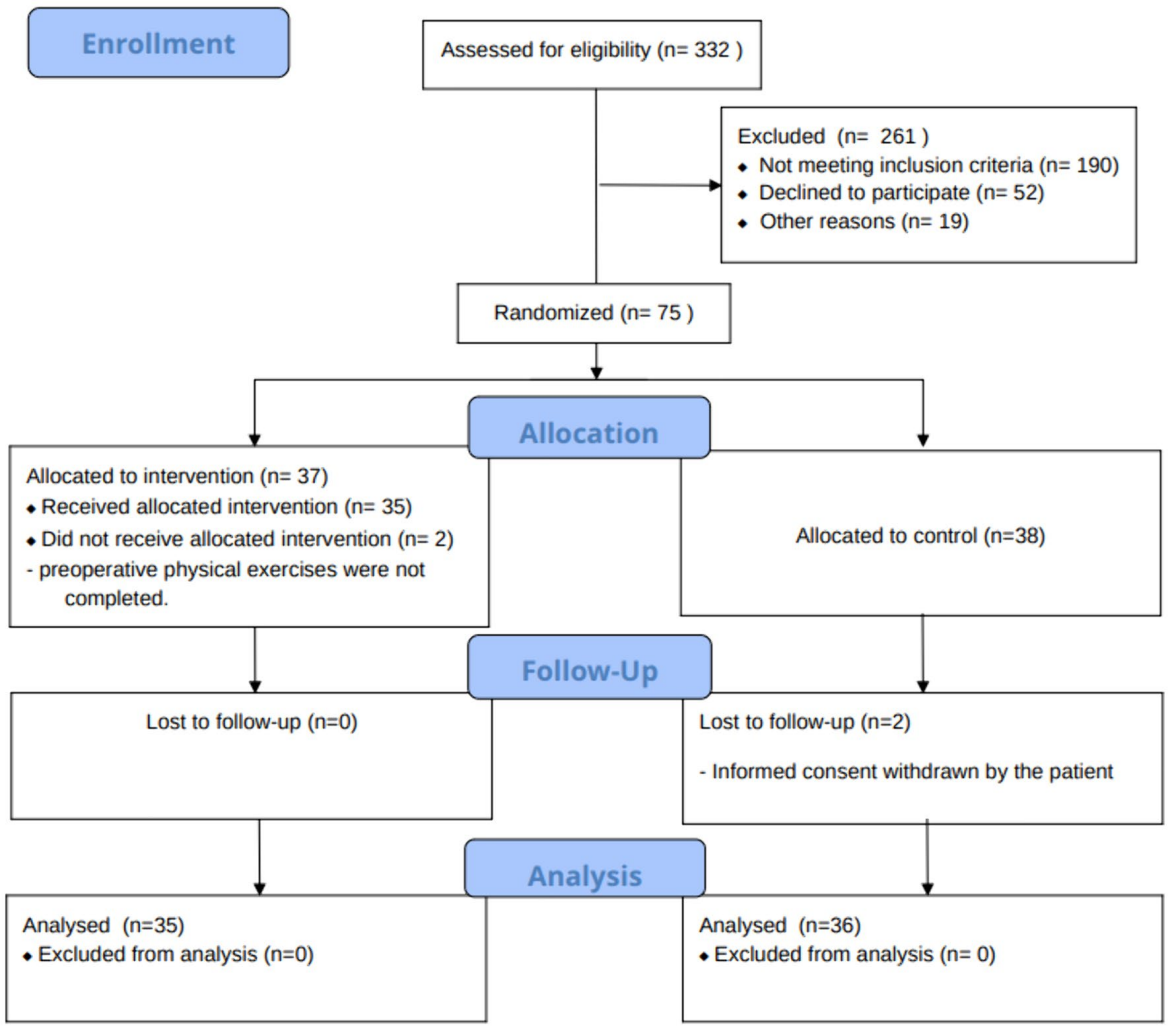
CONSORT flow diagram

In the current study, an interim analysis of 71 patients undergoing colorectal surgery was performed, with 35 assigned to interventional arm and 36 to control arm as shown in CONSORT flow diagram (Fig. 1). Demographic and clinical data are reported in Table 2. Baseline characteristics were comparable in both groups.

**Table 2:** Demographic and baseline characteristics

	Prehabilitation group (*N* = 35)	Control Group (*N* = 36)	*p* value
Total number of patients (71)			
*Gender [N (%)]*			0.886
Male	21 (58.3)	21 (60.0)	
Female	14 (41.7)	15 (40.0)	
Age (years) ± SD	68 ± 8.7	70 ± 9.6	0.329
BMI (Kg/m^2^) ± SD	27.9 ± 5.5	27.8 ± 4.8	0.903
*ASA score [N (%)]*			0.287
1	0	0	
2	16 (47.7)	21 (58.3)	
3	19 (54.3)	15 (41.7)	
CCI ± SD	4.68 ± 1.8	4.86 ± 1.2	0.635
*Coronary disease [N (%)]*			0.217
No	30 (85.7)	34 (94.4)	
Yes	5 (14.3)	2 (5.6)	
*Heart failure [N (%)]*			
No	35 (100)	36 (100)	
Yes	0	0	
*Peripheral vascular disease [N (%)]*			
No	31 (88.57)	33 (91.6)	0.662
Yes	4 (11.43)	3 (8.4)	
*COPD [N (%)]*			
No	34 (97.14)	36 (100)	0.307
Yes	1 (2.86)	0	
*Diabetes mellitus [N (%)]*			
No	29 (82.8)	33 (91.6)	0.265
Yes	6 (17.2)	3 (8.34)	
*Kidney failure [N (%)]*			
No	34 (97.1)	35 (97.2)	0.368
Yes	1 (2.9)	1 (2.8)	
PG-SGA score [N (%)]	A 22 (66.6)	A 27 (75)	0.446
-4-week	B 11 (33.4)	B 9 (25)	
PG-SGA score [N (%)]	A 12 (38.7)	A 15 (48.3)	0.442
-1 week	B 19 (61.3)	B 16 (51.7)	
Preoperative hemoglobin (g/dl) ± SD	13,07 ± 1.7	13,53 ± 1.9	0.308
Preoperative blood glucose level (mg/dl) ± SD	106,34 ± 16.5	104,47 ± 16.6	0.635

*BMI* body mass index, *ASA* American Society of Anaesthesiologists, *CCI* Charlson comorbities index, *COPD* chronic obstructive pulmonary disease, *PG-SGA* patient-generated subjective global assessment

**Fig. 2:**
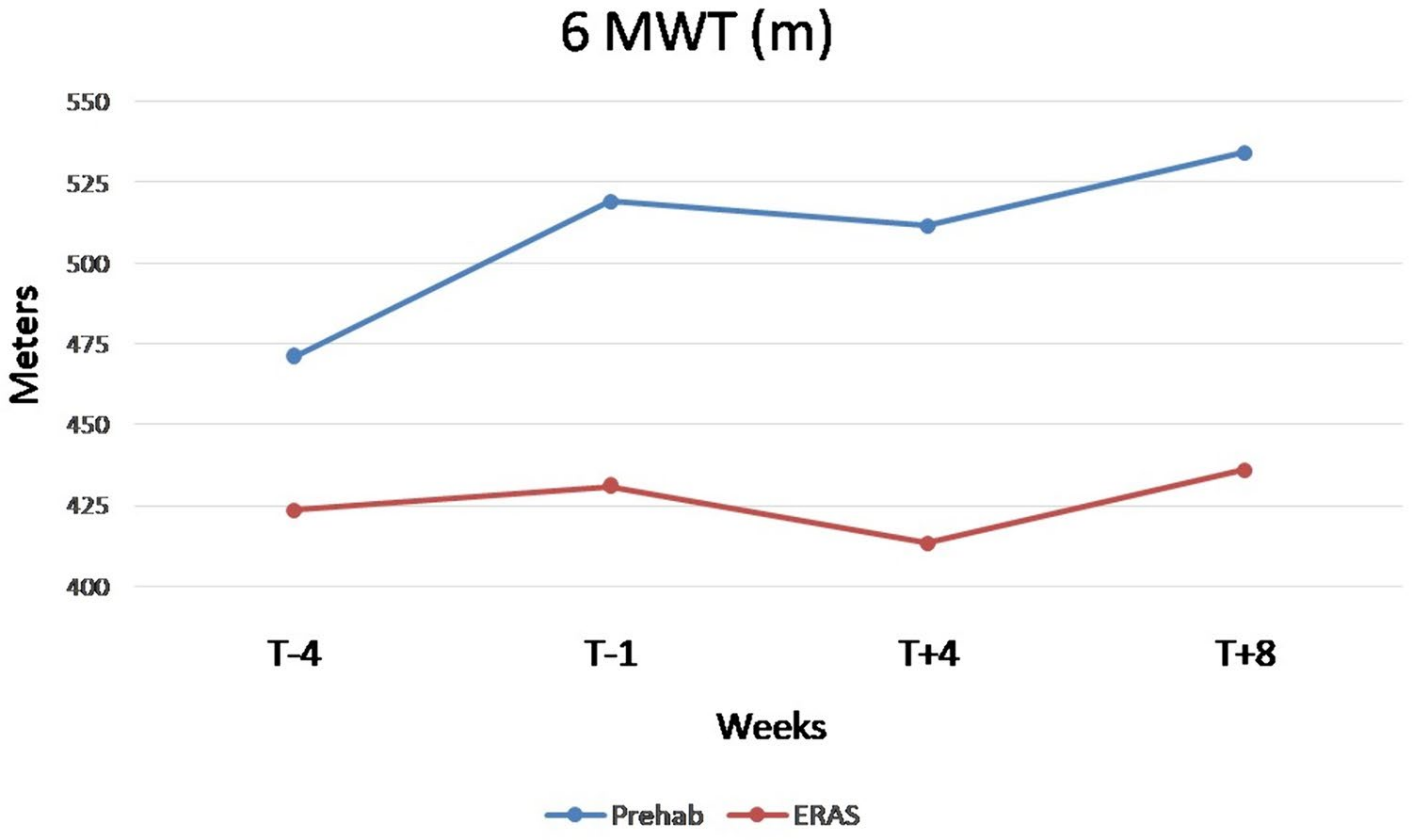
Physical performances at different time points by analyzing the 6MWT

**Fig. 3:**
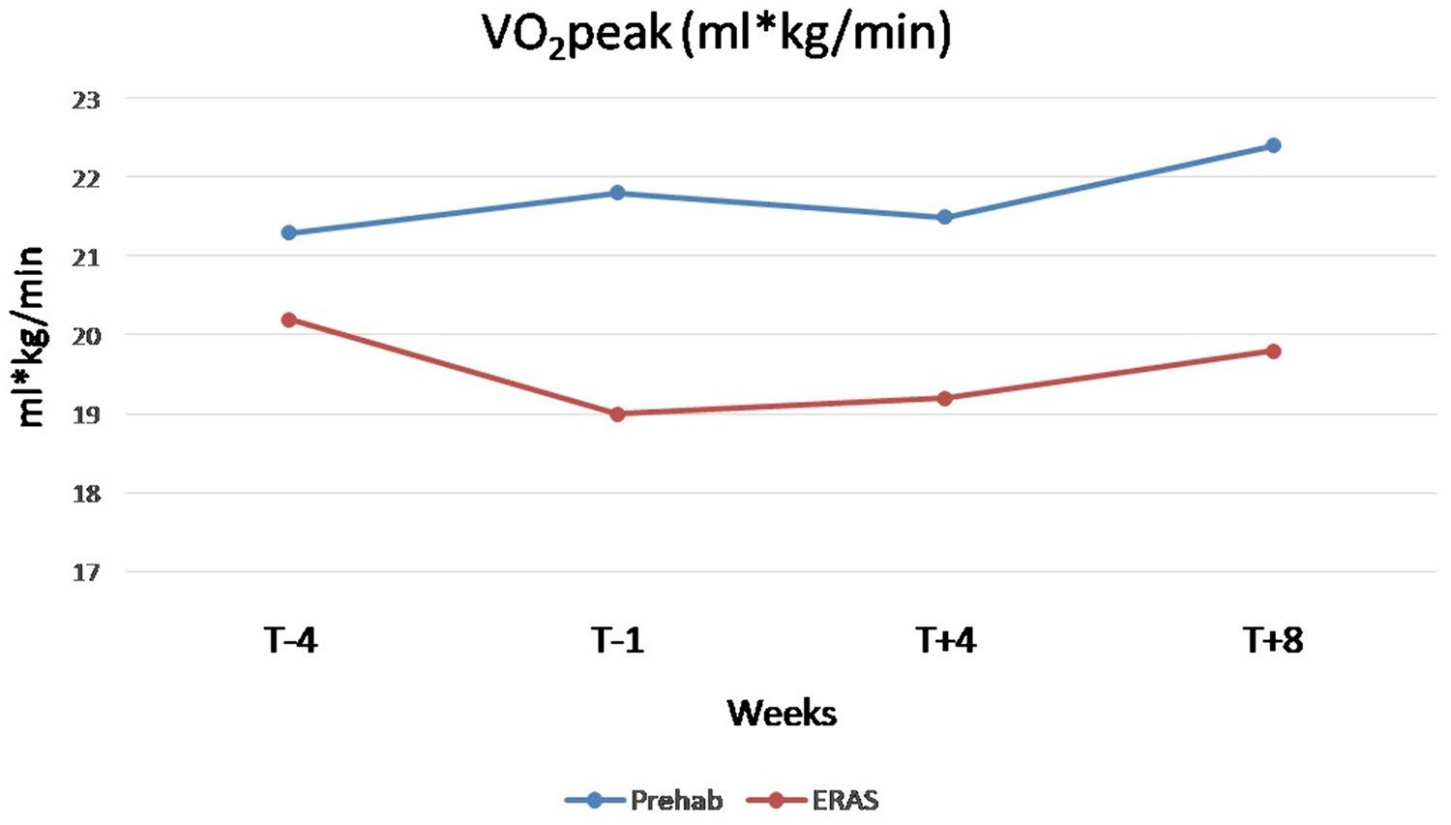
Physical performances at different time points by analyzing the VO2Max

The most commonly performed surgical procedure was right colectomy, no patient underwent a transverse colectomy, and laparoscopy was the predominant approach used (over 94% in both groups). Intra-operative variables are summarized in Table 3. There were no significant differences in both post-operative complications and hospital postoperative length of stay between the two groups [4 days (IQR 2–6) in both groups], as shown in Table 4. The preliminary results revealed that the prehabilitation group demonstrated a significant increase in mean 6MWT distance pre-operatively (T -4 vs. T 0) compared to the control group, with an increase of 96 m (523 ± 24.6 vs. 427 ± 25.3, *p* = 0.01) (Tables 5 and 6). At 4 (T + 4) and 8 (T + 8) weeks, the prehabilitation group maintained significant improvements, with an increase of 103 m (514 ± 89 vs. 411 ± 115, *p* = 0.003) and 90 m (531 ± 82 vs. 441 ± 107, *p* = 0.008), respectively (Tables 7 and 8). Moreover, the physical performances at different time points by analyzing the 6MWT and VO2Max were greater in prehabilitation group compared with the control group, as shown in Figs. 2 and 3. When analyzing the psychometric tests and nutritional assessment we didn’t find any statistically significant differences between the two groups. The Patient-Generated Subjective Global Assessment (PG-SGA) including weight, intake, symptoms, functional status, disease state, metabolic stress and nutritional physical examination at 1 week before surgery did not show any statistically significant differences (*p* = 0.442). Regarding psychometric analysis, only one patient was above the cut off in psychometric screening tests (i.e., GAD-7 and PHQ-9), but he was not sent to the psycho-oncologist as he belonged to the control group.

**Table 3:** Intraoperative variables

Variables	Prehabilitation group (*N* = 35)	Control Group (*N* = 36)	P Value
*Epidural catheter insertion [N (%)]*			
Yes	5 (14.2)	5 (13.8)	0.962
No	30 (85.8)	31 (86.2)	
*Peripheral nerve block [N (%)]*			
Yes	26 (74.2)	23 (63.8)	0.344
No	9 (25.8)	13 (36.2)	
Intraoperative infusions (ml) ± SD	208.8 ± 40.6	221.2 ± 57.2	0.298
*PONV prophylaxis [N (%)]*			
Yes	14 (40.0)	20 (55.5)	0.190
No	21 (60.0)	16 (44.5)	
*Type of surgery [N (%)]*			
Right colectomy (Extended right colectomy)	25 (71.5)	21 (58.5)	0.272
	7 (20.0)	4 (11.1)	
Left colectomy (Extended left colectomy)	6 (15.7)	5 (13.8)	
	0	0	
Transverse colectomy	0	0	
Sigmoidectomy	4 (12.8)	10 (27.7)	
*Surgical approach [N (%)]*			
Laparoscopy	34 (97.2)	34 (94.5)	0.225
Laparotomy	1 (2.8)	0	
Laparoscopy with conversion	0	2 (5.5)	
Other procedure [N (%)]	8 (22.8)	4 (11.1)	0.187
*Abdominal drain [N (%)]*			0.494
Yes	21 (60.0)	19 (52.7)	
No	13 (40.0)	16 (47.3)	
*Postoperative transfusion [N (%)]*			0.539
Yes	2 (5.7)	1 (2.7)	
No	33 (94.3)	35 (97.3)	
*ICU admission [N (%)]*			0.290
Yes	3 (8.6)	1 (2.8)	
No	32 (91.4)	35 (97.2)	
ICU length of stay (day) ± SD	0.4 ± 2.04	0.02 ± 0.16	0.246

*PONV* post-operative nausea and vomiting, *ICU* Intensive care unit

**Table 4:** Measured postoperative variables and outcomes

Variables	Prehabilitation group (*N* = 35)	Control Group (*N* = 36)	*p* value
Oral liquid intake (day) Mean ± SD	1.66 ± 2.05	1.32 ± 0.63	0.356
Solid oral intake (day) Mean ± SD	2.14 ± 2.2	1.94 ± 0.91	0.627
Time to gas canalization (day) Mean ± SD	2.08 ± 0.91	1.8 ± 0.83	0.177
Time to stool canalization (day) Mean ± SD	2.94 ± 1.18	2.97 ± 1.09	0.917
Time to optimal pain control (NRS < 4) (day) Mean ± SD	2.8 ± 1.65	2.54 ± 1.29	0.471
Early mobilization (day) Mean ± SD	2.74 ± 3.22	2.2 ± 0.90	0.340
Fit for discharge (day) Mean ± SD	4.77 ± 4.25	4.14 ± 1.24	0.404
Discharge (day)			0.426
Mean ± SD	5.45 ± 4.61	4.8 ± 1.53	
[median (IQR 25–75)]	4 (2–6)	4 (2–6)	
Clavien- Dindo complications [*N* (%)] total 15/71 (21.1)			
Grade I	2 (5.7)	5 (13.9)	0.565
Grade II	4 (11.4)	2 (5.5)	
Grade IIIa	1 (2.8)	1 (2.8)	
Grade IIIb	0 (0.0)	0 (0.0)	
Grade IV	0 (0.0)	0 (0.0)	
Grade V	0 (0.0)	1 (2,8)	
Reintervention [*N* (%)]	1 (2.8)	2 (5.5)	0.572
30-day hospital re-admission [*N* (%)]	0 (0.0)	1 (2.8)	0.321
30-day mortality [*N* (%)]	0 (0.0)	1 (2.8)	0.321
TNM staging [*N* (%)]			
0	7 (20.0)	4 (11.1)	0.561
I	11 (31.4)	11 (30.5)	
II	8 (22.8)	13 (36.1)	
III	9 (25.7)	8 (22.2)	
IV	0 (0.0)	0 (0.0)	

*NRS* numerical rating scale

**Table 5:** Physical performances at 4 weeks before surgery

Variables	Prehabilitation group (*N* = 35)	Control Group (*N* = 36)	*p* value
6MWD (m)	480 ± 105.2	424 ± 111.9	0.07
VO2 peak (ml/kg/min)	20 (15.5–35.4)	19 (10–45)	0.35
Hand grip test (Kg)	34 ± 12.8	35 ± 11.4	0.82
Sit-to-stand test	13.5 (6–30)	13 (9–21)	0.59

**Table 6:** Physical performances after 4 weeks of prehabilitation before surgery

Variables	Prehabilitation group (*N* = 35)	Control group (*N* = 36)	*p* value
6MWD (m)	523 ± 24.6	427 ± 25.3	**0.01**
VO2 peak (ml/kg/min)	20.4 (14–32.3)	16.9 (10–33)	**0.03**
Hand grip test (kg)	37 ± 16	35 ± 11	0.6
Sit-to-stand test	12.5 (8–31)	12 (8–31)	0.8

**Table 7:** Physical performances at 4 weeks after surgery

Variables	Prehabilitation group (*N* = 35)	Control group (*N* = 36)	*p* value
6MWD (m)	514 ± 89	411 ± 115	**0.003**
VO2 peak (ml/kg/min)	22 ± 5	20 ± 6	0.38
Hand grip test (kg)	33 ± 11	32 ± 10	0.8
Sit-to-stand test	15.5 (8–29)	12 (8–29)	**0.08**

**Table 8:** Physical performances at 8 weeks after surgery

Variables	Prehabilitation group (*N* = 35)	Control group (*N* = 36)	*p*- value
6MWD (m)	531 ± 82	441 ± 107	**0.008**
VO2 peak (ml/kg/min)	21 ± 4	20 ± 5	0.4
Hand grip test (kg)	34 ± 12	33 ± 9	0.8
Sit-to-stand test	18 (12–33)	13 (9–30)	**0.01**

## Discussion

The origins of prehabilitation can be traced back to the early twentieth century, during the Second World War, when L.G. Rowntree published an article on the importance of rehabilitation and prehabilitation in the Journal of the American Medical Association in 1942 [15]. However, it wasn’t until the 1990s that the term “prehabilitation” was coined and the concept gained popularity in the medical community [16]. The first applications were in the field of sports medicine, where athletes engaged in structured exercise and nutrition programs to enhance their performance and prevent injury. The concept was later adapted for use in the surgical setting, with the goal of improving patients’ physical and mental resilience prior to surgery to improve the outcomes and reduce post-operative complications. The use of prehabilitation has since expanded to other areas of medicine, including oncology, cardiology, and pulmonology, as researchers have recognized the potential benefits of improving patients’ health and functional status before undergoing medical interventions [16].

The interventions commonly included physical exercise, nutritional counseling, smoking cessation, and psychosocial support. Previous clinical studies in colorectal surgery have evaluated various outcomes, including physical function, length of hospital stay, postoperative complications, and quality of life [17–20]. Overall, these studies suggest that prehabilitation can improve physical function and reduce postoperative complications in colorectal cancer patients [21, 22], although they were underpowered and heterogenous in terms of the content and delivery setting of the prehabilitation interventions, making it difficult to draw firm conclusions about the most effective components of prehabilitation programs.

In a RCT from Gillis C et al*.* in 2014 [23], the authors found that the 6MWT was significantly higher in the prehabilitation compared with the rehabilitation group at 8 weeks after surgery, without any differences in complication rates and duration of hospital stay.

Recently, the results of the international multicenter PREHAB randomized clinical trial on adult patients with non metastasized colorectal cancer allocated to either 4-week in-hospital supervised multimodal prehabilitation program (high-intensity exercise program 3 times per week, nutritional intervention, psychological support, and smoking cessation) or standard care were published [21]. Despite this trial was prematurely stopped due to the COVID-19 pandemic, it included 251 patients (median age 69 [IQR 60–76] years; 55% male) in the analysis representing the largest RCT to date on colorectal prehabilitation. Primary outcome measures were the number of patients with Comprehensive Complication Index (CCI) score > 20 (i.e., severe complications) and 6MWT 4 weeks postoperatively. The number of severe complications was significantly lower in favor of prehabilitation compared with standard care (21 of 123 [17.1%] vs 38 of 128 [29.7%]; OR 0.47 [95% CI 0.26–0.87]; *p* = 0.02). Also, prehabilitated participants encountered fewer medical complications (e.g., respiratory) vs. controls (15.4% vs. 27.3%; OR 0.48; *p* = 0.02). Four weeks after surgery compared with baseline, 6MWT did not differ significantly between groups (mean difference prehabilitation vs. standard care 15.6 m; *p* = 0.07), although it remained above the baseline level in prehabilitated patients and a greater proportion of patients improved ≥ 20 m (clinically meaningful difference). In addition, 8-week 6MWT differed significantly in favor of the prehabilitation group. Finally, secondary parameters of functional capacity in the postoperative period generally favored prehabilitation compared with standard care [21].

Despite the potential benefits of prehabilitation, there are several challenges associated with implementing prehabilitation programs in clinical practice. One of the main challenges is identifying patients who would benefit from prehabilitation and ensuring that they receive appropriate interventions. This requires close collaboration between surgeons, anesthesiologists, and other members of the healthcare team. Another challenge is the need for resources and infrastructure to support prehabilitation programs. This includes access to exercise facilities, nutritional counseling services, and psychosocial support. In addition, prehabilitation programs require a significant investment of time and effort from patients, which may be difficult for some patients to accommodate.

For this reason, we decided to perform this study in order to analyse the feasibility of a prehabilitation program in surgical patients and the results in terms of physical performance improvement at 4 and 8 weeks after surgery.

At 4 weeks after surgery (T + 4), the prehabilitation group exhibited an average 6MWD that was approximately 100 m higher than the control group, with a statistically significant result. The key aspect is that the trend of increased mean remained consistent between the two groups, at least for the 6MWD. This indicates that the initial prehabilitation has an important effect even post-surgery, resulting in a significant improvement after just 4 weeks. The VO2 peak showed a slight increase but was not statistically significant, as was the case for the sit-to-stand test. This could be attributed to higher efficiency of the cardiorespiratory system, which may not be detectable through statistical measurements but still has a positive impact on the overall aerobic capacity of the patients. The increased physical activity during prehabilitation may have contributed to improving cardiorespiratory endurance and energy efficiency of the system.

Finally, at 8 weeks after surgery (T + 8), the trend remained the same. What differs from T + 4 is the sit-to-stand test, which shows a significant increase. This could be attributed to several factors; during prehabilitation, specific exercises aimed at improving lower limb strength may have positively influenced the patient’s ability to rise from a sitting position. Additionally, the postoperative recovery process associated with prehabilitation may have promoted greater muscle healing and overall strength improvement.

It is important to emphasize that further research is needed to delve into the specific reasons for these improvements and to confirm the results. Exploring physiological changes and adaptive mechanisms associated with prehabilitation could provide a better understanding of the observed benefits in terms of improved aerobic capacity and muscle strength.

There was no difference in postoperative complications and hospital length of stay between the prehabilitation and control groups, which is not surprising considering the power of this study. Patients were cared for within a well established enhanced recovery pathway with almost all patients operated on by minimally invasive surgery, and length of stay was relatively low in both groups. However, this study was not powered to determine the impact of prehabilitation on postoperative outcomes.

## Limitations and strength

Based on preliminary data, about 20% of patients may decline participation due to work commitments, lack of support in the elderly or unwillingness to exercise. Some patients may also fear to delay surgery, although the usual wait at our institution for elective cancer surgery (about 5 weeks) was not prolonged for the participants.

As patients in the study cannot be masked to the intervention, controls may increase their physical activity awaiting for surgery due to the hypothesized potential benefit of prehabilitation. Thus, in order to monitor this potential bias, we planned to objectively assess in both groups the amount of preoperative physical activity using an accelerometer. These data may be helpful for the interpretation of the results.

The cost of the prehabilitation program implementation as well as the indirect costs is still ongoing and therefore it is not included in this preliminary report. Finally, direct (in-hospital) costs were not evaluated as no difference in postoperative complications or ICU stay was detected and all participants followed the same perioperative enhanced recovery pathway as well as surgical technique (i.e., laparoscopy).

The prehabilitation protocol has proven to be feasible and safe and the proposed training was effective in improving exercise functional capacity in surgical patients, due to multidisciplinary involvement.

## Conclusions

The preliminary results of this study indicate that it is feasible to implement a prehabilitation protocol lasting approximately 4 weeks. This protocol appears to yield a significant improvement in the physical performance of patients with colon cancer undergoing elective colorectal resection at 4 and 8 weeks after surgery. However, due to the preliminary nature of these findings and the study’s current power, it remains uncertain whether the proposed intervention can reduce postoperative complications and the duration of hospital stay. The implementation cost of the program and the indirect costs will be assessed upon the completion of patient recruitment.

## Declarations

### Research group

Francesco Bagolini, Luca Bonazza, and Sabrina Pedon (surgeons); Anna Chiara Piva and Cristina Sgarbi (dietitians); Maria Giulia Nanni, Rosangela Caruso, and Luigi Grassi (psycho-oncologists); Gianni Mazzoni (sport doctor); Stefano Volpato (geriatrician, statistical analysis); Emidia Vagnoni (Health economist); Desi Angeli (colorectal cancer case manager).
